# Increased condom use among key populations using oral PrEP in Kenya: results from large scale programmatic surveillance

**DOI:** 10.1186/s12889-022-12639-6

**Published:** 2022-02-14

**Authors:** Griffins O. Manguro, Abednego M. Musau, Daniel K. Were, Soud Tengah, Brian Wakhutu, Jason Reed, Marya Plotkin, Stanley Luchters, Peter Gichangi, Marleen Temmerman

**Affiliations:** 1 International Center for Reproductive Health Kenya, 3rd Avenue Nyali, P.O Box 91109-80103, Mombasa, Kenya; 2 Faculty of Medicine and Health Sciences, Gent University, Gent, Belgium; 3 Jhpiego Kenya, Nairobi, Kenya; 4 Jhpiego, Baltimore, Maryland USA; 5 Institute for Human Development, Aga Khan University, Nairobi, Kenya; 6 International Centre for Reproductive Health, Department of Obstetrics and Gynecology, Ghent University, Ghent, Belgium; 7 School of Population Health and Preventive Medicine, Monash University, Melbourne, Australia; 8 Technical University of Mombasa, Mombasa, Kenya; 9 Department of Public Health and Primary Care, Faculty of Medicine and Health Sciences, Ghent University, Ghent, Belgium; 10 Department of Obstetrics and Gynecology, The Aga Khan University, Nairobi, Kenya

**Keywords:** Pre Exposure Prophylaxis, PrEP, Risk compensation, Female sex workers, Men having sex with men, Sub-Saharan Africa, Kenya

## Abstract

**Background:**

Female sex workers (FSW) and men having sex with men (MSM) in Kenya have high rates of HIV infection. Following a 2015 WHO recommendation, Kenya initiated national scale-up of pre-exposure prophylaxis (PrEP) for all persons at high-risk. Concerns have been raised about PrEP users' potential changes in sexual behaviors such adopting condomless sex and multiple partners as a result of perceived reduction in HIV risk, a phenomenon known as risk compensation. Increased condomless sex may lead to unintended pregnancies and sexually transmitted infections and has been described in research contexts but not in the programmatic setting. This study looks at changes in condom use among FSW and MSM on PrEP through a national a scale-up program.

**Methods:**

Routine program data collected between February 2017 and December 2019 were used to assess changes in condom use during the first three months of PrEP in 80 health facilities supported by a scale-up project, *Jilinde*. The primary outcome was self-reported condom use. Analyses were conducted separately for FSW and for MSM. Log-Binomial Regression with Generalized Estimating Equations was used to compare the incidence proportion (“risk”) of consistent condom use at the month 1, and month 3 visits relative to the initiation visit.

**Results:**

At initiation, 69% of FSW and 65% of MSM reported consistent condom use. At month 3, this rose to 87% for FSW and 91% for MSM. MSM were 24% more likely to report consistent condom use at month 1 (Relative Risk [RR], 1.24, 95% Confidence Interval [CI], 1.18–1.30) and 40% more likely at month 3 (RR, 1.40, 95% CI, 1.33–1.47) compared to at initiation. FSW were 15% more likely to report consistent condom use at the month one visit (RR, 1.15, 95% CI, 1.13–1.17) and 27% more likely to report condom use on the month 3 visit (RR 1.27, 95% CI, 1.24–1.29).

**Conclusion:**

Condom use increased substantially among both FSW and MSM. This may be because oral PrEP was provided as part of a combination prevention strategy that included counseling and condoms but could also be due to the low retention rates among those who initiated.

**Supplementary Information:**

The online version contains supplementary material available at 10.1186/s12889-022-12639-6.

## Background

Following the 2015 World Health Organization (WHO) recommendation [1], the use of oral pre-exposure prophylaxis (PrEP) for HIV prevention is expanding in sub-Saharan Africa. In 2016, the Kenya National AIDS and STI Control Program recommended that people at risk of HIV infection should be provided oral PrEP [2]. Following the recommendations, oral PrEP is offered, and is available to all persons at substantial ongoing risk of HIV infection as part of a package of combination prevention. This includes both the general population and key populations (sex workers, men having sex with men, transgender individuals and people who inject drugs). National scale-up of PrEP began in 2017 [3]; by 2021, PrEP was available in 900 health facilities in Kenya and over 36,000 individuals had initiated PrEP [4].

In Kenya, the estimated prevalence of HIV in 2018 among Female sex workers (FSW) was 29.3 per cent and 18.1 per cent among men having sex with men (MSM), [5] while the prevalence in the general population was 4.9% [5, 6]. Not only is the HIV prevalence among FSW and MSM high, the incidence in both of these populations is also high. In a cohort of MSM in Mombasa, the incidence of HIV was 8 times higher than recorded in the general population for the same period [7, 8]. The last Kenya Modes of Transmission Study conducted by the National AIDS Control Council in 2009, through mathematical modelling, estimated that almost 33% of all recently-acquired HIV infections were attributed to FSW, MSM and people who inject drugs [9]. In Kenya, HIV prevalence in MSM and FSW is associated with older age, lower level of education, having a live-in partner and excessive use of alcohol and recreational drugs [7]. To date, increasing access to services and reducing new HIV infection among FSW and MSM remains a key priority in the fight against HIV [10, 11]. Current interventions are hinged on a peer-led strategy with a balanced focus of biomedical, behavioural and structural interventions [12, 13]. Oral PrEP is provided as part of a combination prevention package [13]. Clinical services to FSW and MSM are provided largely through community-led drop-in centers and outreaches [14].

There are concerns about the possibility of reduced condom use as part of risk compensation (adopting behaviors such as condom less vaginal or anal sex, or multiple sexual partners based on the assumption of protection against HIV) [15, 16]. When used consistently, PrEP reduces an individual's risk of HIV infection by more than 90% [17, 18]. Reduced condom use following PrEP initiation, on the other hand, may increase the risk of unintended pregnancies and other sexually transmitted infections (STIs), both of which are public health priorities. FSW in Kenya, for example, have high rates of unintended pregnancy, low uptake of highly effective contraception, and low rates of dual method use among those seeking to avoid pregnancy [19].

For MSM, findings on changes in condom use vary: a PrEP clinical trial in six countries (Peru, Ecuador, South Africa, Brazil, Thailand, and the USA) reported decreases in condomless sex among those on PrEP [20]. Similarly, follow-up of an MSM cohort previously enrolled in a clinical trial, and an observational study, both in the USA reported decreased condomless sex during one year [21, 22]. In contrast, a systematic review and meta-analysis of 16 observational studies and one open-label trial reported increased condomless sex among MSM on PrEP [23]. For FSW, demonstration projects in Benin, Senegal and South Africa all reported decreases in condomless sex among those on PrEP [24–26]. The same was reported in an observational study in Benin [27].

These research studies report varying changes in condom use among PrEP users. However, less is known about changes in condom use in large scale programs. In the latter settings, PrEP is provided to thousands of people through routine health services. It is anticipated that many people may initiate PrEP due to the widespread availability, and if adherence and risk reduction counselling is inadequate as a result of limited resources, sexual behavior may change, including an increase in condom less sex. Many countries in Africa are transitioning from PrEP demonstration studies to national scale-up. As such, there is need for data on changes in sexual behavior, including condom use, especially among groups such as FSW and MSM who are a key target for most national scale-up programs and who already have high rates of unintended pregnancy and STIs. To this end, we examined changes in self-reported condom use among FSW and MSM using oral PrEP in a national scale-up project in Kenya, between February 2017 and December 2019. We hypothesized that condomless sex would not increase in as much as this is one of the largest oral PrEP scale-up programs in Africa because PrEP is provided as part of a comprehensive combination prevention strategy as recommended by the National AIDS and STI Control Program.

## Methods

### Setting, subjects and service delivery models

We used routine program data to assess changes in condom use during the first three months of PrEP, based on medical records of FSW and MSM who received oral PrEP in health facilities supported by a Bridge-to-scale project, *Jilinde*, in 10 counties in Kenya (Mombasa, Kilifi, Kwale, Taita Taveta, Nairobi, Kiambu, Machakos, Kisumu, Migori and Kisii).

*Jilinde* is a five-year project in Kenya implemented from July 2016 to September 2021, in close collaboration with the Ministry of Health to catalyze PrEP scale-up for FSW, MSM, adolescent girls and young women. PrEP is provided through public, private health facilities, and specialized clinics (drop-in centers) for FSW and MSM. In all health facilities, PrEP is provided as part of a combination prevention package, as outlined in Kenya’s National AIDS and STI Control Program guidelines for HIV/STI programming for key populations, and the National antiretroviral treatment Guidelines [2, 13]. FSW and MSM receive peer education on HIV and STI from trained peer educators (fellow FSW and MSM). Peer educators also distribute condoms, lubricants and information materials, then refer fellow MSM and FSW for clinical services to Jilinde-supported facilities. This model for FSW and MSM comprehensive services has been described elsewhere [13]. The PrEP cascade for all population groups on PrEP through *Jilinde*, which illustrates the process of receiving services from the initial HIV testing, risk screening, initiation and continuation, and identifies gaps at each of these steps, have been presented elsewhere [28].

Clinical services are standard across *Jilinde*-supported sites but most FSW and MSM prefer to receive services through drop-in centers because, in addition to health services, the centers have a dedicated "safe space" for social interaction. At all Jilinde-supported facilities, health providers (HTS counsellors, nurses and clinical officers) provide individual-centered risk-reduction assessment, counselling and testing for HIV. HIV-negative clients undergo behavioural risk screening using a standard national tool, the Risk Assessment Screening Tool, administered by the service providers. Clients who screen positive, or who request oral PrEP undergo a clinical assessment, and are started on PrEP, if eligible. Clients on PrEP attend routine follow-up visits every month. Risk assessment, including assessment for condom use, is carried out during each visit by clinicians. HIV testing is done at initiation, at month 1, month 3 and every third months thereafter, as per national guidelines [2].

### Data sources and data management

We analysed individual client data from the initiation and the first three months visit from 80 health facilities (33 DICs, 38 public and 9 private facilities). We included data for all FSW and MSM who started oral PrEP between February 2017 and December 2019. During this period, *Jilinde* supported 89 health facilities in 10 counties, 80 of which provided services to FSW and/or MSM and are included in the study.

Routine program data were collected using standardized Ministry of Health tools. Data on HIV testing and PrEP screening was entered into national HIV testing services registers. Data on PrEP initiation and follow-up was entered on PrEP encounter forms. After the health care providers entered the data onto the physical forms, the data from the PrEP encounter forms were entered into an encrypted soft copy database (*Jilinde* Data System). Each clinical site submitted a monthly site-level report. PrEP data was also entered into the Kenya Health Information System. Monthly data verification and quarterly data quality audits were conducted to ensure data quality.

As part of the routine service delivery risk assessment at the PrEP initiation visit and during follow-up visits, health care providers asked clients about their condom use. As per the questionnaire, clients were asked if they had “inconsistent or no condom use” in all sexual encounters with each sexual partner in the past 30 days. This was recorded as either yes (to inconsistent or no condom use at some point or at all times) or no (consistent condom use).

### Statistical analysis

The primary outcome was self-reported condom use, which was modelled as a binary variable (yes or no). “Yes” meant that the participant had had condomless sex at any time in the preceding month while “No” meant that the participant had used condoms consistently. The primary predictor variable was time point on oral PrEP (Initiation visit (month 0), month 1 visit, and month 3 visit). Analyses were conducted separately for FSW and for MSM. We used a log-binomial regression analysis to compare the incidence proportion (“risk”) of consistent condom use at the month 1, and month 3 visits relative to the initiation visit, and to generate relative risk (RR). Generalized estimating equations (GEE) were used to account for repeated measurements of consistent condom use. We also estimated association between condom use at any time point and other predictors of condom use within this population. Predictors in the Bivariable analyses were selected a priori*,* based on existing evidence. These included age, marital status, previous history of a STI, and sex under influence of alcohol and drugs. Variables associated with condom use at *p* values of less than 0.05 in bivariable analysis were included in the final multivariable models.

### Ethical oversight

This study was approved by the Kenya Medical Research Institute Scientific Ethics Review Unit (SERU) (approval number Non-KEMRI 601). The John Hopkins Bloomberg School of Public Health issued a non-human subjects’ research determination for the study (IRB No: 000083467).

## Results

### Baseline social and demographic characteristics

Between February 2017 and December 2019, 17,758 FSW and 4,849 MSM were initiated on PrEP at 80 *Jilinde*-supported health facilities. Of those initiated, 22% of the FSW (*n* = 3,791) and 17% of MSM (*n* = 822) revisited the sites to receive PrEP refills and for HIV testing for at least three months. The *Jilinde* PrEP cascade has been described in detail elsewhere [28]. About 95% of the FSW (*n* = 3950) and 97% of the MSM (*n* = 795) received PrEP at drop-in centers. The social and demographic characteristics at PrEP initiation for FSW and MSM included in this analysis are summarized in Table I. The median age was 28 years for FSW (interquartile range [IQR], 23–32 years) and 24 years for MSM (IQR, 21–29 years). About 3% of both FSW (*n* = 95) and MSM (*n* = 22) lived with a partner who was HIV positive. About 79% of FSW (*n* = 2,985) and 83% of MSM (*n* = 682) had a sexual partner whose HIV status was unknown to them and who they considered to be at a high risk of acquiring HIV. Additionally, 6% of FSW (*n* = 236) and 7% of MSM (*n* = 56) reported a recent history (in the past six months) of a sexually transmitted infection.

**Table 1:** Baseline Social, Demographic, and sexual Characteristics of female sex workers (*n* = 3,791) and men having sex with men (*n* = 822) who completed at least three months of PrEP

Variable	FSW (*n* = 3,791) Median (IQR) or Number (percent)	MSM (*n* = 822) Median (IQR) or Number (percent)
Age		
Up to 20	395 (10%)	134 (16%)
21 to 24	783 (21%)	307 (37%)
25 to 30	1,274 (34%)	227 (28%)
31 to 34	560 (15%)	64 (8%)
35 and over	779 (21%)	90 (11%)
Marital status		
Never married	2,379 (63%)	700 (85%)
Married/cohabiting	410 (11%)	82 (10%)
Divorced/separated/widowed	984 (26%)	40 (5%)
Lives with a partner who is HIV positive	95 (3%)	22 (3%)
Self-reported history of infection with a sexually transmitted infections (in the past 6 months)	236 (6%)	56 (7%)
Reports having sex under the influence of alcohol and drugs (Once in the past 30 days)	1,351 (36%)	323 (39%)
Self-reported consistent condom use (in the past 30 days)	2,621 (69%)	531 (65%)
Used post exposure prophylaxis more than once in the past six months before beginning PrEP	173 (5%)	19 (2%)
Recent history of intimate partner violence (IPV) or gender based violence (GBV)	49 (1%)	12 (1%)
Has a sex partner(s) whose HIV status is unknown to them but they consider him/her to be at a high risk of HIV	2,985 (79%)	682 (83%)
Health facility type		
Public/private	201 (5%)	27 (3%)
Drop-in Centre	3,590 (95%)	795 (97%)
Geographical cluster		
Coast	1,263 (33%)	210 (26%)
Lake	1,224 (32%)	367 (45%)
Nairobi	1,304 (34%)	245 (30%)

### Condom use among FSW

**Fig. 1:**
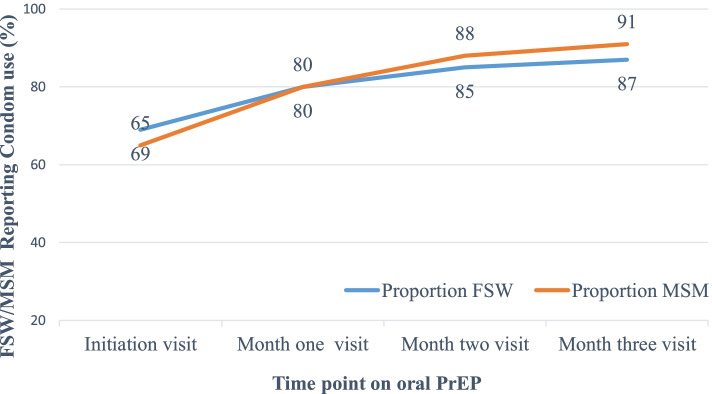
Condom use among FSW and MSM from initiation of PrEP to Month Three

At PrEP initiation, 69% of FSW who were included in the analyses (*n* = 2621) reported consistent condom use. Among those who did not continue with PrEP up to the month 3 visit and were not included in the analysis, 67% (*n* = 9276) reported using condoms consistently. In the population included in the analysis, the proportion increased to 80% on the month 1 visit and to 87% on the month 3 visit. In comparison to PrEP initiation, FSW were 15% more likely to report consistent condom use at the month one visit (Relative Risk [RR], 1.15, 95% Confidence Interval [CI] 1.13–1.17) and 27% more likely to report condom use on the month 3 visit (RR 1.27, 95% CI, 1.24–1.29). Fig. 1 shows the change in self-reported condom use during the first three months of PrEP for both FSW and MSM.

Older FSW were more likely to report condom use at any PrEP visit; FSW aged 31 years and above were 9% more likely to report condom use compared to those aged 20 years and below (RR, 1.09, 95% CI, 1.04–1.14). Similarly, FSW with a live-in partner (those who were married or cohabiting) were more likely to report condom use compared to those who did not have a live-in partner (RR, 1.08, 95% CI, 1.05–1.12). FSW who received services at the DIC were also more likely to report condom use compared to those who received services at private and public health facilities (RR, 1.43, 95% CI, 1.30–1.58). On the contrary, FSW who reported a recent history of a STI in the previous six months and those who reported engaging in sex while under the influence of alcohol and recreational drugs were less likely to report condom use (RR, 0.89, 95% CI, 0.84–0.94 and RR, 0.90, 95% CI, 0.87–0.92 respectively). In multivariable analyses, condom use at the month three visit was still greater than at PrEP initiation (ARR, 1.26, 95% CI, and 1.23–1.28). Table 2 presents bivariable and multivariable analyses of factors associated with reporting condom use during the first three months of oral PrEP for MSM.

**Table 2:** Characteristics Associated with Self-Reported Condom Use among Female Sex Workers Who Completed at Least Three Months of oral PrEP (*n* = 3791)

**Variable**	**Bivariable Analysis**		**Multivariable Analysis**	
	**Relative Risk (95% CI)**	***p*-Value**	**Relative Risk (95% CI)**	***p*-Value**
Time point on oral PrEP				
Initiation visit	Reference		Reference	
Month one visit	1.15 (1.13–1.17)	0.001	1.14 (1.12–1.16)	0.001
Month two visit	1.22 (1.20–1.25)	0.001	1.22 (1.19–1.24)	0.001
Month three visit	1.27 (1.24–1.29)	0.001	1.26 (1.23–1.28)	0.001
Age				
20 years and below	Reference			
21 to 24 years	0.99 (0.95–1.04)	0.796	0.91 (0.68–1.23)	0.554
25 to 30 years	1.01 (0.97–1.06)	0.582	1.03 (0.77–1.39)	0.825
31 to 34 years	1.09 (1.04–1.14)	0.001	1.19 (0.87–1.63)	0.270
35 years and over	1.09 (1.04–1.14)	0.001	1.39 (1.01–1.94)	0.045
Marital status				
Never married	Reference			
Married or cohabiting	1.08 (1.05–1.12)	0.001	1.67 (1.34–2.15)	0.001
Divorced/Separated/Widowed	1.00 (0.97–1.03)	0.898	0.90 (0.76–1.06)	0.200
Self-reported recent history of infection with STI (in the past six months)	0.89 (0.84–0.94)	0.001	0.47 (0.37–0.60)	0.001
Reports having sex under the influence of alcohol and recreational drugs	0.90 (0.87–0.92)	0.001	0.67 (0.48–0.77)	0.001
Type of health facility				
Public/Private	Reference		Reference	
Drop-in Centre	1.43 (1.30–1.58)	0.001	1.47 (1.35–1.55)	0.001
Geographical region of residence				
Coast	Reference			
Lake	0.86 (0.83–0.89)	0.001	0.91 (0.88–0.94)	0.001
Nairobi	1.08 (1.06–1.11)	0.001	1.05 (1.03–1.08)	0.001

^a^ Multivariable models included Age, Marital Status, History of infection with an STI, Having sex under the influence of alcohol and drugs, type of health facility and geographical region of residence

### Condom use among MSM

During the PrEP initiation visit, 65% of MSM (*n* = 531) who were included in these analyses reported consistently using condoms in the previous month. When we compared them to the MSM who did not come for the month 3 visit and were not included in the analysis, 77% (*n* = 3105) used condoms consistently at PrEP initiation. The proportion of MSM who used condoms consistently increased to 80% on the month 1 visit and to 91% on the month 3 visit. MSM were 24% more likely to report using condoms consistently on the month 1 visit (RR, 1.24, 95% CI, 1.18–1.30) and 40% more likely at the month 3 visit (RR, 1.40, 95% CI, 1.33–1.47) compared to the PrEP initiation visit.

MSM who were between 21 and 24 years and those between 25 and 30 years were less likely to report using condoms compared to those aged 20 years and below, who were the reference category (RR, 0.93, 95% CI, 0.88–098 and RR, 0.88, 0.82–0.94 respectively). Similarly, MSM who reported engaging in sex while under the influence of alcohol and drugs were 13% less likely to report using condoms than those who did not (RR, 0.87, 95% CI 0.83–0.92). There was, however, no association between condoms use and being married or cohabiting (RR, 1.04, 0.98–1.12), reporting a recent history of a STI (RR, 1.00, 95% CI, 0.92–1.08), and receiving services at DICs (RR, 1.02, 95% CI, 0.86–1.22). In multivariable analysis, condom use was still significantly higher at the month 3 visit compared to the PrEP initiation visit (ARR, 95% CI, 1.33–1.47). Table 3 presents bivariable and multivariable analyses of factors associated with reporting condom use during the first three months of oral PrEP for MSM.

**Table 3:** Characteristics Associated with Self-Reported Condom Use among Men Having Sex with Men who Completed at Least Three Months of Oral PrEP (*n* = 822)

**Variable**	**Bivariable Analysis**		**Multivariable Analysis**	
	**Relative Risk (95% CI)**	***p*-Value**	**Relative Risk** **(95% CI)**	***p*-value**
Time point on oral PrEP				
Initiation visit	Reference		Reference	
Month one visit	1.24 (1.18–1.30)	0.001	1.23 (1.17–1.30)	0.001
Month two visit	1.36 (1.29–1.43)	0.001	1.35 (1.29–1.42)	0.001
Month three visit	1.40 (1.33–1.47)	0.001	1.40 (1.33–1.47)	0.001
Age				
20 years and below	Reference			
21 to 24 years	0.93 (0.88–0.98)	0.010	0.56 (0.34–0.93)	0.026
25 to 30 years	0.88 (0.82–0.94)	0.001	0.44 (0.26–0.75)	0.002
31 to 34 years	0.93 (0.85–1.01)	0.086	0.56 (0.30–1.04)	0.066
35 years and over	0.88 (0.80–0.97)	0.009	0.49 (0.26–0.90)	0.022
Marital status				
Never married	Reference			
Married/cohabiting	1.04 (0.98–1.12)	0.157		
Divorced/Separated/Widowed	0.92 (0.79–1.07)	0.297		
Self-reported recent history of infection with STI (in the past six months)	1.00 (0.92–1.08)	0.905		
Reports having sex under the influence of alcohol and recreational drugs	0.87 (0.83–0.92)	0.001	0.98 (0.93–1.02)	0.271
Type of health facility				
Public/Private	Reference			
Drop-in Centre	1.02 (0.86–1.22)	0.768		
Geographical region of residence				
Coast	Reference			
Lake	1.12 (1.04–1.20)	0.002	1.22 (1.15–1.30)	0.001
Nairobi	1.28 (1.20–1.37)	0.001	1.17 (1.10–1.26)	0.001

^b^ The multivariable model included age, having Sex under influence of alcohol and/or recreational drugs, and the geographical region of sex work

## Discussion

We describe self-reported condom use from the initiation visit to three months of PrEP use for FSW and MSM receiving PrEP through routine health services in Kenya. To the best of our knowledge, this paper is the first in SSA to explore condom use among new users of oral PrEP in the programmatic setting for KP. At the month 3 visit, the proportion of FSW who reported using condoms consistently increased by 18 percentage points and that of MSM by 26 percentage points.

The findings of increased self-reported condom use reported here are consistent with PrEP demonstration projects for FSW and MSM in other parts of Africa [24, 25]. These findings suggest that even in the setting of providing PrEP through routine health care services as part of national scale-up, FSW and MSM did not reduce their use of condoms.

Increased use of condoms in both FSW and MSM may be explained by the extensive risk-reduction counseling that PrEP clients receive at initiation and during each follow-up visit, coupled with the widespread availability of condoms to FSW and MSM through the national programme. These findings suggest that routine use of PrEP will not result in reduced condom use as part of risk compensation among MSM and FSW, especially when service providers offering PrEP through routine, non-research settings are adequately trained and supported, and when PrEP is offered as part of a combination prevention package supported by robust national systems, including adequate access to condoms, as was the case in *Jilind*e. Secondly, a cross-sectional survey among FSW and MSM at PrEP facilities in South Africa reported high condom use (80% at the last time of sexual intercourse) among current PrEP users, and low condom use among those who discontinued PrEP [29]. This may imply that FSW and MSM who continue to use PrEP in non-research settings have innately strong reasons to continue and adhere to condoms and other HIV preventive strategies. As a result, condom use reduction is less likely in this population, and instead, greater focus should be directed to those who commence and quit PrEP. This is supported by further analyses that looked at changes in condom use among a larger population of FSW (8628) and MSM (2285) who were on oral PrEP at month one (Table 1 and Table 2 respectively in the Supplementary file). Both tables show that condom use still increased among all FSW and MSM who were on PrEP at the end of month 1. A third explanation for increased condom use may be because taking an antiviral pill on a daily basis serves as a daily reminder of the risk of HIV, particularly for persons who already have higher risks of acquiring HIV such as FSW and MSM. Consistent reminders of ongoing risk have been demonstrated to increase sex planning and positively modify sexual behavior including using condoms more and having less partners [30]. However, it is also possible that the reported increase in condom use is due to a social desirability bias, especially given that data on condom use was collected via a clinical form completed by the clinician during each visit. FSW and MSM may report that they use condoms because they do not want to disappoint the clinician or interviewer, or because they are afraid that PrEP may be withheld if they report that they do not use condoms. Such desirability bias have been reported in studies where there are significant differences between self-reported condom use and biomedical markers of condomless sex like prostatic-specific antigen (PSA) and Y-chromosome detection [25].

The rates in condom use reported at the PrEP initiation visit in both MSM and FSW were not markedly different from those observed from routine, monthly-collected program monitoring data by implementing partners in Kenya and through periodic surveys conducted by the National AIDS and STD Control Program. Kenya’s 2018 data estimates that 92% of FSW and 77% of MSM used condoms at the last sexual intercourse and 73% of FSW and 69% of MSM used condoms consistently in the last month [31, 32]. Both our data and the national data demonstrate that in general, consistent condom use among FSW and MSM in Kenya is higher than that reported for other countries in Africa [33–36]. Similarly, condom use among FSW and MSM is higher compared to use among HIV discordant couples and the general population in Kenya [5, 37]. Programs providing services to FSW and MSM are coordinated by the National AIDS and STI Control Program which routinely collects data on FSW and MSM services and outcomes in order to respond to emerging needs. Condoms and lubricants are distributed to FSW and MSM free of charge through peer educators and at the drop-in centers. In 2018, almost 90% of FSW and 80% of MSM were served by a peer educator and 74% of FSW and 68% of MSM visited the drop-in centers at least once every three months [32]. Having a dedicated national program for FSW and MSM and integrating PrEP for FSW and MSM through such programs likely optimizes oral PrEP outcomes by building synergy with other HIV prevention interventions such as peer education. Further, in addition to the biomedical effect of PrEP in reducing new infections, providing PrEP to FSW and MSM through such programs may increase their engagement with complimentary services [21].

Our findings are subject to several limitations. First, condom use was self-reported and could be subject to social desirability bias as discussed above. Secondly, we used clinic service data from routine program implementation and our data was not drawn from a research study designed to respond to the question of condom use among PrEP users. As such, data on important predictors of condom use such as such as urban/rural living, education level, awareness of partner's HIV status, pregnancy desire for FSW's, and receptiveness/assertiveness for MSM's was not available because it is not part of routinely collected data. Studies also have strict controls on protocols and data management which is not available for program data. However, we believe that the routine quality assessments provided adequate checks to ensure data accuracy. Third, our data is limited to the first three months of PrEP and we may not be able to extrapolate or describe trends thereafter. Finally, our study population comprised of FSW and MSM who initiated PrEP and continued taking PrEP for three months. This population could be considered self-selective as only 22% of FSW and 17% of MSM continued taking PrEP up to the third month, and there may be significant differences in risk-compensation between those who continued with PrEP and those who did not. We however did not have data on condom use at subsequent months for FSW and MSM who discontinued PrEP.

Despite the limitations, our study presents important information on early changes in condom use among FSW and MSM on oral PrEP during scale-up. As indicated, this may be the first presentation on potential changes in condom use among FSW and MSM receiving PrEP during national roll-out in a resource-limited setting. From the perspective of a policy planner, the fact that we used patient record data collected through routine clinical services and our participants received standard care could also be considered a strength. These findings therefore present the most accurate case scenario of what would be expected during PrEP scale-up for key populations in other parts of Africa.

## Conclusion

In conclusion, the findings from this programmatic evaluation suggest that risk compensation did not occur in the first 3 months of PrEP use among FSW and MSM in Kenya in the context of routine provision of care. However, there is need for more long-term follow-up data to observe changes beyond three months as PrEP normalizes as a HIV prevention intervention.

## Supplementary Information

**Additional file 1.** Further analysis.

## Data Availability

The datasets used and/or analysed during the current study are available from the corresponding author on reasonable request.

## Abbreviations

CI: Confidence Interval
DIC: Drop-in centre
FSW: Female sex workers
HTS: HIV testing services
IQR: Interquartile Range
KEMRI: Kenya Medical Research Institute
KHIS: Kenya Health Information system
KP: Key populations
MoH: Ministry of Health
MSM: Men having sex with men
NASCOP: National AIDS and STI Control Program
PrEP: Pre exposure prophylaxis
PSA: Prostate Specific Antigen
RAST: Risk Assessment Screening Tool
RR: Relative Risk
SERU: Science and Ethics Unit
