# Supplementary material for: Increased condom use among key populations using oral PrEP in Kenya: results from large scale programmatic surveillance
Source: BMC Public Health. 2022 Feb 14;22:304. doi: 10.1186/s12889-022-12639-6 (PMC8842980; doi:10.1186/s12889-022-12639-6)
Supplement: Supplementary file 1 — Additional file 1. Further analysis. [file 12889_2022_12639_MOESM1_ESM.docx]

**Supplementary File: Further analysis**

The tables below show analyses for FSW (n=8628) and MSM (2285) who completed only one month. For FSW, there was still an increase in condom use at month one (ARR, 2.04, 95% CI 1.94-2.16), slightly higher than what we report ARR, 1.14, 95% CI 1.12-1.16. Similarly, for MSM, there was an increase in condom use at month one, ARR, 2.52, 95% CI 2.20-2.88, slightly higher than what we report, ARR 1.23, 95% CI, 1.17-1.30.

**Table 1: Characteristics Associated with Self-Reported Condom Use among Female Sex Workers who completed at least one month of oral PrEP (n=8628).**

|  | **Bivariable Analysis** | | **Multivariable Analysis** | |
| --- | --- | --- | --- | --- |
| **Variable** | **Relative Risk (95% CI)** | **p-Value** | **Relative Risk (95% CI)** | **p-Value** |
| Time point on oral PrEP |  |  |  |  |
| Initiation visit | Reference |  | Reference |  |
| Month one visit | 1.18 (1.17-1.20) | 0.001 | 2.04 (1.94-2.16) | 0.001 |
| Age |  |  |  |  |
| 20 years and below | Reference |  |  |  |
| 21 to 24 years | 1.03 (0.99-1.08) | 0.129 | 1.09 (0.92-1.28) | 0.316 |
| 25 to 30 years | 1.07 (1.03-1.12) | 0.001 | 1.27 (1.07-1.50) | 0.005 |
| 31 to 34 years | 1.13 (1.09-1.18) | 0.001 | 1.49 (1.24-1.78) | 0.001 |
| 35 years and over | 1.19 (1.14-1.24) | 0.001 | 1.93 (1.59-2.35) | 0.001 |
| Marital status |  |  |  |  |
| Never married | Reference |  |  |  |
| Married or cohabiting | 1.12 (1.09-1.15) | 0.001 | 1.67 (1.42-1.95) | 0.001 |
| Divorced/Separated/Widowed | 1.07 (1.05-1.09) | 0.001 | 1.13 (1.01-1.26) | 0.037 |
| Self-reported recent history of infection with STI (in the past six months) | 0.89 (0.86-0.93) | 0.001 | 0.59 (0.51-0.69) | 0.001 |
| Reports having sex under the influence of alcohol and recreational drugs | 0.82 (0.80-0.84) | 0.001 | 0.44 (0.40-0.48) | 0.001 |
| Type of health facility |  |  |  |  |
| Public/Private | Reference |  | Reference |  |
| Drop-in Centre | 1.33 (1.27-1.40) | 0.001 | 3.2 (2.77-3.71) | 0.001 |
| Geographical region of |  |  |  |  |
| Coast | Reference |  |  |  |
| Lake | 1.02 (0.99-1.04) | 0.780 | 1.10 (0.99-1.22) | 0.069 |
| Nairobi | 0.88 (0.86-0.91) | 0.001 | 0.53 (0.47-0.59) | 0.001 |

**Table 2: Characteristics Associated with Self-Reported Condom Use among Men Having Sex with Men who completed one month on Oral PrEP (n=2285).**

|  | **Bivariable Analysis** | | **Multivariable Analysis** | |
| --- | --- | --- | --- | --- |
| **Variable** | **Relative Risk (95% CI)** | **p-Value** | **Relative Risk**  **(95% CI)** | **p-value** |
| Time point on oral PrEP |  |  |  |  |
| Initiation visit | Reference |  | Reference |  |
| Month one visit | 1.18 (1.15-1.21) | 0.001 | 2.52 (2.20-2.88) | 0.001 |
| Age |  |  |  |  |
| 20 years and below | Reference |  |  |  |
| 21 to 24 years | 0.96 (0.92-1.00) | 0.068 | 0.76 (0.55-1.04) | 0.084 |
| 25 to 30 years | 0.94 (0.89-0.98) | 0.007 | 0.73 (0.52-1.02) | 0.064 |
| 31 to 34 years | 0.96 (0.91-1.02) | 0.166 | 0.87 (0.58-1.29) | 0.482 |
| 35 years and over | 0.94 (0.88-1.00) | 0.042 | 0.69 (0.45-1.07) | 0.100 |
| Marital status |  |  |  |  |
| Never married | Reference |  |  |  |
| Married/cohabiting | 0.99 (0.94-1.04) | 0.657 | 1.21 (0.86-1.72) | 0.267 |
| Divorced/Separated/Widowed | 0.96 (0.88-1.05) | 0.415 | 1.24 (0.78-1.98) | 0.361 |
| Self-reported recent history of infection with STI (in the past six months) | 0.93 (0.88-0.99) | 0.024 | 0.73 (0.54-0.99) | 0.048 |
| Reports having sex under the influence of alcohol and recreational drugs | 0.86 (0.82-0.88) | 0.001 | 0.44 (0.36-0.52) | 0.001 |
| Type of health facility |  |  |  |  |
| Public/Private | Reference |  |  |  |
| Drop-in Centre | 1.20 (1.05-1.38) | 0.007 | 2.91 (1.70-4.99) | 0.001 |
| Geographical region of residence |  |  |  |  |
| Coast | Reference |  |  |  |
| Lake | 0.83 (0.80-0.87) | 0.001 | 0.28 (0.22-0.36) | 0.001 |
| Nairobi | 0.86 (0.84-0.89) | 0.001 | 0.31 (0.25-0.39) | 0.001 |
